# Why low-dose aspirin persists in recurrent pregnancy loss without APS: cognitive bias and system design

**DOI:** 10.1016/j.xagr.2025.100602

**Published:** 2026-01-04

**Authors:** Kentaro Iga, Takeshi Murakoshi, Hiroshi Adachi

**Affiliations:** 1 Division of Perinatology, Fetal Diagnosis and Therapy, Maternal and Perinatal Care Center, Seirei Hamamatsu General Hospital, Hamamatsu, Shizuoka, Japan (Iga, Murakoshi, and Adachi); 2 Center for Medical Education, Nagoya University Graduate School of Medicine, Nagoya, Aichi, Japan (Iga)

**Keywords:** recurrent miscarriage, low-dose aspirin, antiphospholipid syndrome, cognitive bias, therapeutic illusion, ethics, reproductive medicine

## Abstract

Low-dose aspirin (LDA) is widely prescribed for recurrent pregnancy loss (RPL) even when antiphospholipid syndrome (APS) is absent, despite high-level evidence showing no improvement in live-birth rate in such cases. In Japan and elsewhere, this empirical use persists across fertility and obstetric settings, suggesting a behavioral and systemic rather than purely evidentiary problem. This commentary interprets the persistence of LDA through the lens of cognitive bias and institutional design. At the cognitive level, action bias—the instinct to intervene when outcomes are uncertain—provides a sense of control for both patient and clinician after miscarriage. The availability heuristic further normalizes use because LDA is already familiar as prophylaxis for pre-eclampsia, and professional conformity (social proof) reinforces its legitimacy. Once started, status-quo bias and loss aversion make discontinuation difficult, as both clinician and patient fear regret if another loss occurs. Systemic structures amplify these biases. In Japan, time-intensive counseling is rarely reimbursed, whereas prescriptions are; ambiguous phrasing in domestic reports (“may consider aspirin”) subtly encourages action over restraint. Discontinuity between fertility and obstetric care promotes pharmacologic reassurance in place of relational continuity. Together, these conditions transform an evidence-neutral intervention into a psychologically and institutionally self-reinforcing default. Recognizing this pattern reframes empirical LDA as a system phenomenon rather than individual error. Practical countermeasures include bias-awareness education, explicit “do-not-start” criteria limiting LDA to APS-confirmed cases, audit and feedback cycles, and shared decision aids that display live-birth data from randomized trials. Above all, continuity-based reassurance—scheduled follow-up and early-pregnancy access without unnecessary medication—can restore alignment between evidence and empathy. Understanding the cognitive architecture of empirical LDA use enables reproductive medicine to redesign its environment so that good science and good care no longer compete. Recognizing these dynamics can help clinicians and institutions redesign care environments that support evidence-based restraint without diminishing empathy.

Low-dose aspirin is indicated in recurrent pregnancy loss only when antiphospholipid syndrome is confirmed. In women without APS, randomized trials show that aspirin—alone or combined with heparin—does not improve live-birth rates.^1^, ^2^, ^3^, ^4^, ^5^ Yet empirical use persists in many settings, particularly in Japan. This persistence reflects not a lack of knowledge but the predictable influence of cognitive bias, professional culture, and institutional design on clinical decision-making. This pattern, while described here in Japan, reflects a universal challenge in reproductive medicine—how clinicians manage uncertainty and hope when evidence is limited.

The first and most powerful driver is action bias—the natural tendency to favor intervention over inaction when outcomes are uncertain. In the emotionally charged setting of pregnancy loss, both patient and clinician experience helplessness. Prescribing low-dose aspirin can momentarily restore a sense of agency, even when clinical benefit is implausible. This pattern exemplifies the therapeutic illusion—the conflation of emotional relief with medical efficacy^6^ —a bias that can subtly transform empathy into over-treatment.

The availability heuristic further amplifies this pattern. Because low-dose aspirin is legitimately used for preeclampsia prevention,^7^ it feels familiar, safe, and almost routine. That familiarity can distort risk perception, making off-label use for recurrent loss appear intuitively reasonable. As cognitive psychology has long shown, clinicians—like all humans—rely on vivid and easily recalled examples rather than statistical probabilities, turning familiarity into a subtle cognitive trap.

Professional culture adds social proof—the pressure to conform to perceived norms. When most peers prescribe aspirin, abstaining can feel deviant or even negligent, especially in emotionally charged situations such as pregnancy loss. Without regular audit feedback, prevalence itself begins to masquerade as evidence. Over time, this quiet conformity embeds unproven therapy into the community’s default behavior, transforming culture into a sustaining mechanism of low-value care.

Institutional ambiguity reinforces the cycle. Permissive language in national guidelines and governmental reports—phrases such as “consider aspirin”—can make intervention appear safer than restraint. In Japan, reproductive-medicine guidance describes infertility treatment and assisted reproduction in detail but rarely addresses low-dose aspirin in recurrent pregnancy loss. This silence allows interpretive drift across clinical boundaries. When guidance does not clearly specify ‘do-not-start’ criteria for non-APS RPL, aspirin can become a de facto default. Clinicians then fill policy gaps with personal judgment and peer imitation rather than evidence.

In Japan, time-intensive counseling after miscarriage is rarely reimbursed, whereas prescriptions and diagnostic tests are. This economic asymmetry shapes behavior: pharmacologic reassurance is quicker, billable, and culturally legible as “doing something,” while continuity-based reassurance is labor-intensive and financially invisible. Ambiguous phrasing in national reports—for example, “may consider aspirin”—further tilts clinicians toward action in the face of uncertainty. Broad, clinic-level interpretations of “recurrent pregnancy loss” can expand the treated population while lowering the prior probability of benefit, a pattern characteristic of low-value care. Recognizing these intertwined economic and linguistic incentives reframes empirical LDA not as personal error but as the predictable outcome of misaligned system rewards—precisely the kind of target that quality-improvement initiatives can address.

Trust dynamics in fragmented care also play a role. Many women transition between fertility centers and obstetric clinics, and physicians uncertain of follow-up may prescribe something tangible to sustain rapport. This behavior reflects the broader phenomenon of medical reassurance—the use of benign interventions or explanations to relieve anxiety even when clinical outcomes remain unchanged.^8^ As described in primary care for non-specific conditions, reassurance can easily become pharmacologic rather than relational. When continuity is lost, presence is replaced by prescription.

Once started, low-dose aspirin becomes difficult to stop. Status-quo bias and loss aversion discourage discontinuation: both patient and clinician fear regret if another miscarriage follows cessation.^9^ Over time, this shared aversion crystallizes into a self-reinforcing narrative—“we always give aspirin in RPL.” What begins as a protective gesture becomes an inherited routine, detached from evidence yet sustained by culture.

These cognitive forces form a closed loop. Uncertainty triggers action; perceived safety and social imitation normalize it; ambiguity and discontinuity sustain it; and status-quo bias locks it in place. What results is not a failure of competence or compassion, but a structural expression of how cognition operates under uncertainty—a system reacting exactly as designed.

Some clinics even refuse chorionic villus sampling or minor procedures if patients are taking LDA, citing bleeding risk—despite inconsistent evidence that LDA increases significant bleeding. Such reactions, though inconsistent with data, reflect small acts of professional resistance: efforts to regain moral clarity in a system blurred by uncertainty.

The ethical cost is false reassurance. Low-dose aspirin conveys a sense of protection it cannot deliver, diverting time and trust away from higher-value care such as APS testing, genetic counseling, or psychosocial support. Each consultation spent sustaining an unproven therapy carries an opportunity cost—what might have been done instead. Informed autonomy is also eroded: under emotional strain, patients are more vulnerable to framing effects and more likely to accept treatments described as “preventive” than those labeled “unproven.”

For some clinicians, requests for aspirin can feel like a subtle test of trust—declining may be perceived as withdrawal of care. When patients insist despite explanation, clinicians may experience a quiet erosion of commitment: a sense that evidence-based restraint is misread as indifference. In this way, empirical aspirin may not only be ineffective but relationally harmful, shifting the encounter from shared understanding to negotiated compliance.

“Aspirin is cheap and safe; why deny hope?” Hope should be truthful. When success without treatment is misattributed to medication, false reassurance replaces genuine accompaniment, diverting attention from higher-value care and weakening future decision-making.

“What about ‘borderline’ or non-criteria APS?” Diagnostic uncertainty should prompt verification, not assumption—rigorous APS testing or research enrollment rather than routine off-label therapy. The ethical alternative to empirical prescribing is transparent uncertainty combined with structured follow-up.

“Patients want something.” What they often seek is not a pill but presence. Action can be relational: scheduled early-pregnancy access, clear return instructions, and written no-treatment plans are concrete acts of care that preserve trust and autonomy without medicalizing grief.

Solutions require both cognitive and structural redesign. Professional education should explicitly address action bias, availability heuristic, social proof, and status-quo bias—transforming unexamined reflexes into reflective awareness. Clinics can implement do-not-start criteria limiting LDA to APS-confirmed cases, reinforced by periodic audit and feedback. Electronic order systems can prompt clinicians to justify LDA prescriptions in non-APS indications, gradually reversing the default from action to reflection.

Implementation begins with awareness, not accusation. Electronic health records could include a soft “do-not-start” prompt that asks clinicians to confirm antiphospholipid syndrome before ordering aspirin. When a prescription is entered for a non-APS case, a brief rationale field can invite reflection—a pause before action. Regular audits, perhaps quarterly, can track the frequency of off-label LDA use, while peer comparison can gently reshape local norms. Decision aids should make the evidence visible: a single page showing that live-birth rates near 50% without empirical therapy can dissolve the illusion of benefit. Finally, structured continuity should replace symbolic medication—scheduled post-loss consultations, clear early-pregnancy access, and familiar staff who accompany patients across pregnancies. Together, these modest, low-cost interventions can turn evidence into habit and empathy into system design.

At the patient interface, shared decision aids summarizing trial results^2^, ^3^, ^4^ can make the absence of benefit visible and credible. When patients understand that live-birth rates reach 50% irrespective of intervention,^10^ pharmacologic reassurance loses its appeal.

Above all, continuity-based reassurance should replace pharmacologic reassurance. Scheduled follow-up and early access in subsequent pregnancies offer stability and hope without unnecessary medication. Clinically, longitudinal follow-up after miscarriage—without empirical treatment—has been shown to provide reassurance and stability, consistent with cohort data reporting overall live-birth rates near 50%, and approximately 60% among those who conceive again, regardless of aspirin use^10^.

The continued use of LDA in non-APS recurrent pregnancy loss reflects the predictable interplay between cognitive bias and systemic uncertainty. Recognizing this pattern shifts the focus from individual blame to environmental design. By embedding bias awareness, transparency, and relational continuity into daily practice, reproductive medicine can sustain both scientific integrity and human reassurance.

## Ethics approval and consent

This article does not involve human participants or identifiable data; therefore, institutional review board approval was not required.

## Declaration of generative AI and AI-assisted technologies

During the preparation of this work, the authors used ChatGPT (OpenAI, San Francisco, CA) for language refinement and formatting. After using this tool, the authors reviewed and edited the content as needed and take full responsibility for the content of the published article.

## CRediT authorship contribution statement

**Kentaro Iga:** Writing – review & editing, Writing – original draft, Resources, Project administration, Methodology, Investigation, Formal analysis, Data curation, Conceptualization. **Takeshi Murakoshi:** Supervision. **Hiroshi Adachi:** Supervision.
